# Social learning is critical to breastfeeding success: Evidence from rural Namibian pastoralists

**DOI:** 10.1093/emph/eoaf030

**Published:** 2025-10-21

**Authors:** Brooke A Scelza

**Affiliations:** UCLA Department of Anthropology, University of California, Los Angeles (UCLA), Los Angeles, CA 90095-1553, USA

**Keywords:** breastfeeding, evolutionary mismatch, assisted reproduction, cooperative breeding

## Abstract

**Background and objectives:**

Lactation is one of the defining features of mammals, yet many humans struggle with breastfeeding. One reason for this is that humans are unique among mammals in the degree of learning and support that they require to breastfeed successfully. Despite this, we know little about how social learning impacts breastfeeding, particularly outside the influence of biomedical systems.

**Methodology:**

Qualitative and systematic interviews were conducted with 128 Namibian women on infant feeding norms and practices. Structured statements were analyzed with cultural consensus analysis to determine whether a single cultural model exists and to identify variance in individual cultural competencies.

**Results:**

Cultural consensus analysis revealed a single cultural model for breastfeeding, with strong and consistent norms and a significant role for social learning. Both learning and instinct were invoked in women’s responses, speaking to the necessary and expected role of intensive support in the early postpartum period. Women also noted steep learning curves and clear expectations about infant feeding, which led to nearly universal breastfeeding and clear paths for troubleshooting difficulties.

**Conclusions and implications:**

The breastfeeding support that Himba mothers receive is part of the legacy of assisted reproduction in humans. However, the features of intensive teaching and learning shown here are lacking in western models of infant feeding and postpartum care. These data suggest that protracted breastfeeding difficulties may result from a mismatch between the evolved socioecology of breastfeeding and current norms and practices that hinder social learning and impair support pathways.

## BACKGROUND

One of the defining features of mammals is the feeding of mother’s milk to their young. Because of this, we often view breastfeeding as not only “natural” but innate. However, numerous studies in the health and social sciences have detailed women’s struggles with breastfeeding, with a high percentage of mothers unable to meet their breastfeeding goals [1, 2]. This has led health organizations like the WHO, UNICEF, and the American Academy of Pediatrics to make improving breastfeeding rates a core part of their institutional goals and initiatives; however, breastfeeding outcomes still fall below target rates [3]. These well-documented struggles are seemingly at odds with our mammalian legacy, which would have required successful breastfeeding to survive, raising the question: why do women have so many difficulties enacting a behavior that is critical to our survival and reproductive success?

One common response within the biomedical community is that western culture and medicine have disrupted processes of imprinting and early bonding [4–6]. This has led to interventions like facilitating immediate skin-to-skin contact, and rooming-in with infants in the hospital [7]. In addition, social scientists have pointed to institutional barriers like hospital policies, the lack of paid parental leave, inadequate breastfeeding promotion, and insufficient support as key to understanding why breastfeeding success rates are not higher [1, 7, 8]. There is general agreement, however, that there has been a loss of intergenerational knowledge transmission due to increasing medicalization of birth and breastfeeding, compounded by the structural barriers that make breastfeeding more challenging, particularly among minority communities [9]. Underlying all these issues is the fact that humans are unique among mammals in the degree of learning that is required to breastfeed successfully [10].

Learning to breastfeed appears to be part of our primate legacy but is accentuated in humans. Whereas other mammals initiate feeding with little effort or struggle, primates display a mix of instinct and learning around infant feeding. Primate mothers who were raised in isolation are unable to nurse [11] and captive apes who have exposure to other ape mothers are more successful at feeding their infants [12]. In humans, these difficulties are compounded by anatomical differences in breast shape and suckling, and by secondary altriciality, which hinders infant control over latch and positioning [10]. For humans, as with non-human primates, social learning is critical to overcoming these hurdles and breastfeeding successfully. As Volk explains, “our ancestors found a stable and effective solution by reliably living in large groups that promoted alloparental care of breastfeeding mothers through direct, informational, and socio-emotional support” [10].

Several features of breastfeeding make it more likely to be learned socially, rather than through trial-and-error individual learning (Laland 2004; Boyd and Richerson 1985/1988). First, infant feeding is a costly arena to make mistakes in as it is tightly tied to survival and infant health is highly sensitive. Second, breastfeeding is a complex task, both in terms of decisions around feeding (when to supplement, how often to feed) and the act of feeding itself (obtaining proper latch, avoiding and treating injuries to the breast). In addition, novices are more likely to use social learning, particularly when there are plenty of adult models, indicating that social learning may be particularly important for primiparous mothers. The few studies available that apply social learning theory to breastfeeding support these premises. One key study in rural Tanzania looked at knowledge about WHO recommended infant and young child feeding practices, showing that members of the same ethnic group and women in close proximity tend to have similar beliefs [13]. Several other studies have also shown evidence of social transmission of infant feeding behavior [14–16].

In the public health literature, there is now widespread appreciation of the need for instruction in breastfeeding, with aid available from a wide variety of professional and lay sources [8, 17]. This help comes mainly in two forms: in the hospital immediately following birth, or as an acute response after problems have arisen [18, 19]. The former is most famously illustrated through the Baby-Friendly Hospital Initiative, jointly sponsored by the WHO and UNICEF, which began in the early 1990s. The initiative promotes successful breastfeeding with a 10-step strategy, several of which are focused on teaching and learning. These include, “ensuring staff have sufficient knowledge, competence, and skills to support breastfeeding,” and supporting mothers to “manage common difficulties,” and to “recognize and respond to their infants’ cues for feeding” [20]. While this initiative successfully increased rates of establishment of breastfeeding, there are significant hurdles to in-hospital care. One is that hospital-based breastfeeding efforts occur in the immediate postpartum period, before the onset of lactogenesis II, when “milk comes in.” While the length of a typical hospital stay varies around the world, one recent survey of 71 countries reported 34% had average stays of 2 days or less, while 62% had stays ≤ 3 days [21]. In addition, even in baby-friendly hospitals lactation support tends to be designated to nurses, who often lack training specific to lactation [22, 23], and is just one of their many duties, making consistent, repetitive support impractical [20]. Finally, only a small percentage of women give birth in baby-friendly hospitals [24]. Highlighting these issues, a recent global review calls for better, multilevel interventions, including breastfeeding support over a longer timespan, from pregnancy through the postpartum [25].

This pattern stands in stark contrast to the ethnographic literature on the postpartum period, where many studies describe an intensive period for rest and recuperation after birth with reductions in physical labor, special foods that promote lactation, and regular attendance and support for new mother by kin, including help with breastfeeding [26, 27]. While several of these studies highlight the importance of learning to breastfeed, and the role of elder kin and midwives as instructors, they provide little detail on the process of teaching and learning. Relatedly, we know little about how social norms might be fostering breastfeeding success by setting expectations, providing resources for help, or transmitting ideologies about infant feeding. In this study, a mixed-methods, anthropological approach is used to illuminate the norms and practices of early breastfeeding in a rural, Namibian population where exclusive breastfeeding is widespread. The results highlight the important role that social learning plays in breastfeeding success and suggest that intensive teaching and support are critical to the unique socioecology of human reproduction. Furthermore, the results underscore the ways that breastfeeding learning and practice in much of the world has changed, resulting in a critical disjuncture between ancestral and contemporary practices.

## METHODOLOGY

This study was conducted with Himba pastoralists (*n* = 127) living in the Kunene District of Namibia, close to the Angolan border. Himba are semi-nomadic, but in this region they tend to maintain a relatively permanent compound where they spend much of the year and maintain gardens, as well as herding cattle and goats. They practice double-descent, maintaining close ties with maternal kin, and women generally return to their natal home before each birth, typically staying for several months postpartum [28]. Increasingly women are giving birth in the hospital, but their hospital stays are short, and even those going to hospital have the pre- and postpartum periods with maternal kin [29]. Breastfeeding is nearly universal, although women do sometimes supplement with goat milk when infants are separated or when mothers perceive insufficient supply. Formula is generally unavailable to women living in rural areas and is expensive for all so is rarely used. Women generally breastfeed for several years, typically weaning when they are about halfway through their next pregnancy.

Data were collected in 14 villages outside the regional capital of Opuwo (avg. distance 61.6 km, range 8.4–166 km) during the dry season in 2024. All respondents were women, ranging in age from 15 to 83 (*n* = 127). Most women in the sample were of reproductive age (94.5%) and had given birth at least once (86.6%), but nulliparous women and women who were postmenopausal were both included. This facilitated an understanding of widespread norms and enabled the inclusion of perspectives by women as both mothers and allomothers. A sub-sample of 15 women were asked about their first birth experiences. These women were chosen based on their openness during the initial part of the interview so may represent a biased sample. However, previous more systematic questioning about birth experiences showed similar responses [29]. This work was approved by the UCLA Institutional Review Board (IRB-10-0238) and the Namibian Department of Home Affairs (#0011124007046) and was conducted in collaboration with the Hizetjitwa Indigenous People’s Organization (HIPO). Oral consent was given by all participants and the heads of each household where interviews were conducted also provided consent.

### Data collection

A semi-structured interview format was used. First, participants were presented with 21 “true/false” binary statements about infant feeding and the postpartum period. These statements were generated using ethnographic interviews on birth and breastfeeding conducted over 14 years of previous fieldwork in this population [26, 28, 29]. The statements were designed to elicit general cultural knowledge and beliefs rather than individual opinions or preferences and were therefore focused on birth norms rather than personal experience. Statements were translated from English to *Otjiherero*, backtranslated by a fluent *Otjiherero* speaker for verification, and piloted with a small group (*n* = 10) of Himba in a community ~15 km from Opuwo. Following this, to compare norms with personal experience, a series of follow-up questions were asked to clarify and expand upon responses to the binary statements and to learn more about women’s birth, breastfeeding, and caretaking experiences. All interviews were conducted in *Otjiherero* by the author and a translator so that questions and follow-ups could be addressed during the interviews as needed.

### Consensus analysis

Cultural consensus analysis (CCA) was used to determine whether there is a set of shared beliefs about a particular domain across a group of respondents [30]. CCA determines whether there is evidence for a single cultural model for a given domain, identifies the culturally “correct” responses to statements about that domain, and assesses the level of cultural competence survey respondents have about the domain. In other words, CCA allows researchers to “gain access to the socially transmitted and shared information pool” [31].

A general Condorcet model with hierarchical Bayesian inference was used to generate the degree of consensus for each item as well as individual competency scores for all respondents. Item difficulty is accounted for in the model and a comparison of variance dispersion index between real data and model-predicted data indicated that the inclusion of all items was appropriate. $\hat{R}$ values were used to assess model convergence (all $\hat{R}$ values < 1.05). Differences in competency scores by age, parity, education level, and distance to town were also assessed. Analysis was done in R [32] via rStudio [33] using CCTpack [34], which permits analysis of data with missing responses. Additional packages used for data cleaning and visualization included *tidyverse, janitor, and patchwork* [35–37].

## RESULTS

**Figure 1:**
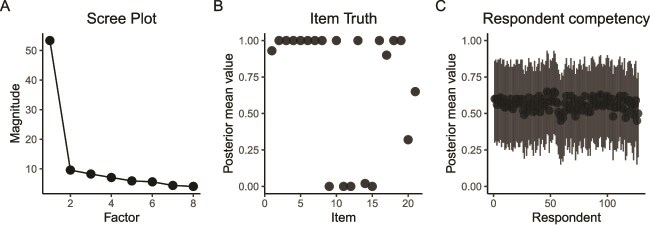
Consensus indicators. (A) Scree plot showing a components analysis of the number of consensus groups in the respondent set. Here, a single cultural group is depicted. (B) Item truths show the consensus-based “correct” answers to each statement (1 = agree, 0 = disagree), with almost all items showing a strong shared understanding. (C) Variation in respondent competency across all statements. Competency is measured as the probability (0–1) that an individual responds correctly to any given question. Bars represent 95% high-density intervals.

A sample of 127 women completed the 21-item consensus survey (2661 responses, 6 missing values). The CCA showed strong cultural consensus across the survey. A scree plot depicts strong respondent agreement and a single cultural model (Fig. 1A) with most item scores close to either 0 or 1 (Fig. 1B), and strong consistency in respondent competency scores (Fig. 1C).

**Figure 2:**
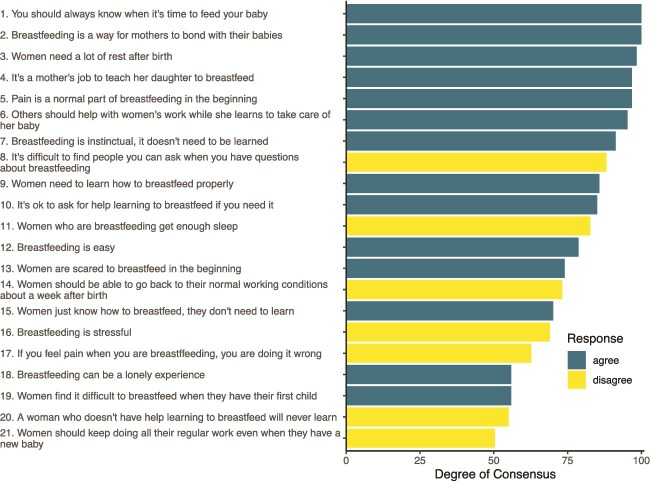
Level of agreement on consensus survey statements. Statements are ordered from most to least agreement. Negatively valanced responses (majority disagree) are in lighter (yellow) hue.

Of the 21 items, 12 had > 75% consensus among respondents and all but 4 had > 60% consensus (Fig. 2). There was very strong agreement about women’s need for rest and recuperation after birth (Items 3, 6, and 14), breastfeeding as a conduit to maternal–infant bonding (Item 2), and the role of advice and support related to breastfeeding (Items 4, 6, 8, and 10). There was also strong consensus about breastfeeding difficulties, like pain (Item 5), loneliness (Item 18), and fear (Item 13).

A number of statements addressed the degree of learning that is needed (Fig. 2), and statements about instinct vs learning generated mixed results. For example, 91% agreed with the statement “Breastfeeding is instinctual, it doesn’t need to be learned,” but 86% reported that “Women need to learn how to breastfeed properly” with 57% of respondents agreeing with both statements. However, there was strong consensus across the statements about help, with 94% believing it’s OK to ask for help if you need it, 94% agreeing that it is a mother’s job to teach her daughter to breastfeed, and 100% disagreeing that it is difficult to find people to ask if you have questions about breastfeeding.

When asked about difficulties breastfeeding, slightly more than half (55.9%) reported that it is difficult to breastfeed your first child, although in response to a more general question that was not specific to parity, 78.7% of women reported that breastfeeding was easy. However, this differed by parity, with 92% of the highest parity women (>3 births) agreeing, compared to 65% of nulliparous and 67% of women who had 1–3 births. In addition, only 31% of women reported that breastfeeding was stressful.

More specifically, women were asked about pain during breastfeeding. There was strong consensus (97%) that pain is a normal part of breastfeeding. Most women (62%) disagreed with the statement, “If you feel pain when you are breastfeeding, you are doing it wrong,” signaling normalization of pain, particularly in the early days of breastfeeding. This sentiment was strongest among women of the highest parity (Supplementary Materials).

**Figure 3:**
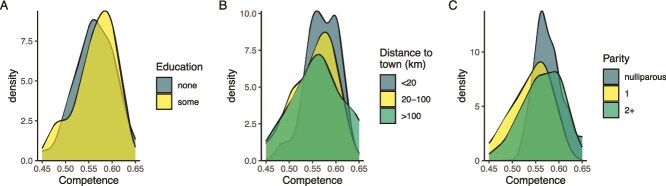
Density plots depicting variation in respondent competency. Competency levels broken out by (A) level of education, (B) distance to the regional capital of Opuwo, and (C) number of children.

Overall, there was little variation in competency scores by either education level, distance from town, or parity, indicating that norms about breastfeeding are widely shared and persistent (Fig. 3). Agreement within items was also fairly uniform; however, there were a few notable exceptions (Supplementary Materials). Fewer nulliparous women than their parous counterparts said that breastfeeding was easy (65% vs 81%) and more believed that, “women just know how to breastfeed, they don’t need to learn,” (94% vs 67%). Women who had some schooling were less likely to say that you should keep doing all your regular work after giving birth than those with no schooling (33% vs 54%), as were women who lived closer to town (33%) compared to their more distant-living counterparts (63%–64%).

Follow-up questions and clarifications to the consensus survey revealed some additional information about the process of teaching and learning. Many women reported staying with their mother, or another close female kin member for weeks to months after a birth, in line with previous work on postnatal propinquity in this population [28]. This person slept in their hut with them, providing 24-hour assistance, and helped with many household tasks like cooking, washing, and collecting wood and water. Among the sub-sample of 15 women who were asked about their first birth experiences all had a key helper (73% listed their mothers, 20% other maternal kin, and 6% paternal kin) and 80% reported some direct teaching about breastfeeding. They described their mothers teaching them how to hold their infants, how to position the baby on the breast, when and how often to feed, and how to co-sleep safely. Reports on these kinds of assistance were provided even by women who said they had no trouble learning to breastfeed.

## DISCUSSION

These data provide strong support for a single cultural model of breastfeeding among Himba women, one which emphasizes the importance of both learning and support. These data help to explain why Himba women have nearly universal breastfeeding success. Understanding why Himba women succeed at breastfeeding can also help us to decipher the evolutionary conundrum that so many women with different cultural models are unable to meet their breastfeeding goals as the data speak to a mismatch between ancestral breastfeeding conditions and the current biomedically focused environment many women in the west encounter.

### Breastfeeding: instinct or learned behavior?

Consensus analysis showed a distinct set of shared beliefs around breastfeeding, but embedded within this cultural model was some ambiguity about whether breastfeeding is an instinctual or a learned behavior. The seemingly conflicting results could result from confusion (e.g. poorly worded statements) or social desirability bias (the tendency to answer “true” to all statements). The former would be unlikely to result in such uniform responses, as confusion is more likely to result in equivocal response rates. The latter also seems improbable, as on average respondents answered “disagree” for 7 of the 21 statements (min = 1, max = 11). Rather, qualitative data collected during the interview indicate that these answers represent a nuanced understanding of how learning and instinct intersect. One woman explained her response to that statement by saying, “It’s instinctual, but you need help.” A more specific example comes from a statement about feeding. One hundred percent of respondents agreed with the statement, “You should always know when it’s time to feed your baby.” However, in follow-up questions when women were asked about the early days of breastfeeding and the help they received, one of their most common recollections was that in the early weeks after a birth their mothers, who would sleep in the hut with them and their infant, would wake them at night to tell them to feed their babies. The expectation here seems to be that you “just know” how to breastfeed *because* you are surrounded by a support system who will guide you through the process.

### How social learning shapes the breastfeeding experience

For Himba women, learning to breastfeed is part of a larger complex of perinatal norms that emphasize rest, recuperation, and support [26]. Particularly after a first birth, but after most subsequent births as well, women relocate to live with close female kin, who provide instrumental, informational, and emotional support. Even those who give birth in hospital, which is increasingly common, they tend to travel with their mothers for the birth and return to her compound for the postpartum period. As one woman recounts, “My mother stayed with me. She woke me up to breastfeed, told me not to breastfeed lying down. She helped me wash the baby. She cooked for me. She talked to me if I was scared.” Similarly, elder women were asked about the kinds of help that mothers provide their daughters, and they also mentioned a combination of direct instruction and advice:

*Put the nipple in the baby’s mouth properly.*

*Don’t breastfeed lying down.*

*Clean the breast before feeding.*

*Don’t let the baby sleep too long without feeding.*

*Don’t let the baby cry too much. Pick it up. Feed it.*

*Don’t jostle the baby around. Be careful.*

This intensive period of support means that women have help with almost every feeding. Combined with breastfeeding on demand at a high frequency, this likely results in a steep learning curve.

Most women reported that they had pain and difficulty with feeding in the first few days after birth, but that this resolved quickly. They received both specific, instructional support on how to get the baby to latch, as well as reassurance that what they were experiencing was normal. Importantly, this included normalization of pain, with the vast majority of respondents agreeing with the statement, “Pain is a normal part of breastfeeding.” Maybe more importantly, most believed that feeling pain was not a signal that you were doing something wrong. One woman explained, “My mother told me to keep putting the baby on the breast. She told me it’s paining because it’s the first time. Just keep going and it will get better.” As another woman put it, “My mother told me, ‘…that’s the war of the woman, that breastfeeding pain’.” In addition, while many agreed with the statement “women are scared to breastfeed in the beginning” relatively few believed that breastfeeding was stressful. One notable difference was that women with many children (>3) believed that breastfeeding was easy (91%), while those with fewer were less likely to agree with this statement (64%–67%), suggesting that difficulties tend to be experienced earlier on in women’s reproductive careers. In sum, the set of strongly shared beliefs in this culture include not only a clear ideology about what breastfeeding will be like (including fear and pain) but an expectation that you will have the support you need to persevere. The result is that, while some supplementation occurs, all the women in the sample breastfed for extended periods.

These data also show that norms about breastfeeding are in place before women give birth for the first time. In general, nulliparous women in the sample responded very similarly to their parous counterparts (Fig. 3C). This includes strong beliefs about the need for learning and that help is available. They also appear to have realistic expectations about what breastfeeding will be like. Similar to their parous counterparts, the majority believe women are scared to breastfeed in the beginning (77%), and like their parous counterparts, believing that breastfeeding can be a lonely experience (47%). All nulliparous women reported believing that pain is normal when beginning breastfeeding (100%) and understand that this does not mean they are doing something wrong (65%).

It is critical to note that having a set of shared norms about what breastfeeding will be like is not the same as “learning by observation.” These data suggest that it is not merely being exposed to other breastfeeding women that matters, it is specific preparation about what to expect and direct teaching that are necessary to help women succeed. Most of the first-time mothers in our sample reported having some struggles with breastfeeding in the beginning, including learning to latch and knowing how to hold the baby properly. Despite growing up viewing breastfeeding regularly, they still required some direct teaching to master the skill—but importantly the internalized cultural norms they had about breastfeeding prepared them for this.

### Could evolutionary mismatch explain discrepancies in breastfeeding success?

The cultural mileau of breastfeeding for Himba women centers on the twin pillars of support and learning. Both support and learning takes place within a period of intensive rest and recuperation, with breastfeeding occurring frequently and on-demand. This sets up an ideal environment for breastfeeding success. Women have many opportunities to learn in the early days after birth, with help at every feeding if needed, and they are able to focus solely on their own recovery and caring for their infant because others take over everyday tasks like cooking, collecting wood and water, and animal husbandry. They are knowledgeable about the kinds of hurdles they are likely to encounter, and they know that they can ask for and expect help to overcome them.

This socioecology, including a higher rate of home births, extensive skin-to-skin contact, co-sleeping, long periods of postpartum recovery, and little separation between mother and infant after birth, differs greatly from the one that most women in the west experience after giving birth. In the immediate postpartum, while women are still in the hospital, breastfeeding support may be available, but it is often intermittent and comes from multiple nurses as they rotate shifts [22, 25]. In addition, postpartum hospital stays are quite short, typically 24–48 hours in the USA, and most of this help is occurring before women’s milk has come in [21]. Once home, women rarely have full-time support or lactation coaching available, although these services are more available in some European countries [38, 39]. In the USA, most workers continue to lack access to any paid maternity leave, particularly those in the lowest income brackets [40]. Others are juggling their own recovery and learning with maintaining the household and caring for older children [41].

The lack of proper training and support can hinder breastfeeding success. Breastfeeding frequency and efficiency of milk withdrawal are the two most important factors impacting milk volume [42]. Problems with latch affect both as ineffective latch directly hinders milk transfer and also leads to pain for the mother, further hindering milk production and breastfeeding frequency [43]. Women then are told they have an insufficient supply, which they often interpret as a physiological deficiency rather than a fixable impediment [8, 44]. This cycle is compounded when pediatric interventions include formula supplementation, which can further dwindle supply [45, 46], as well as with inadequate knowledge (by mothers and providers) about signs of infant hunger, leading to perceptions of insufficient supply [8]. The result is that the most commonly cited reasons for stopping breastfeeding, pain and problems with latch and perceived insufficient supply, both could be ameliorated by better, more consistent training and support [18, 46].

Added to the logistical hurdles, women also often have poor information about what breastfeeding will be like or how to get help if they need it [42]. In the effort to promote breastfeeding over formula-feeding, public health campaigns have highlighted breastfeeding as “natural,” and “beautiful” [47, 48]. This may not only further misconceptions about breastfeeding being instinctual rather than learned behavior, it sets women up to feel like they have failed or are doing something wrong if they experience pain, fear, or anxiety [44, 49, 50]. However, there are some contexts where use of terms like “natural” can be empowering for breastfeeding mothers. This has been shown among African Americans where a return to breastfeeding is an act of cultural reclamation and empowerment [51]. Increasingly, mothers turn to social media for information, advice, and camaraderie [52]. This can be a helpful substitute for deficits in localized, personal support but is unlikely to provide the same kind of directed teaching and learning that Himba women receive.

## CONCLUSIONS AND IMPLICATIONS

Humans have a long legacy of assisted reproduction. As cooperative breeders, there is widespread evidence that mothers receive various forms of assistance with childcare, from infancy through adolescence [53]. This includes a long legacy of assisted birth and support during the postpartum [27]. These data suggest that another important element of the cooperative breeding system in humans is a strong social learning model of breastfeeding. Learning is so ingrained in the Himba cultural model of infant feeding that it appears to be built into women’s understanding “knowing” how to breastfeed. Its inclusion helps to explain how humans have been able to navigate breastfeeding difficulties in the past, and its breakdown can help us to understand why those difficulties are more persistent in much of the world today.

There is broad consensus in the public health community that social support is critical to breastfeeding success, and that new policies and procedures are needed to provide mothers with more opportunities for instruction, advice, and care [54]. Programs like the Baby-Friendly Hospital Initiative and in the USA, improved coverage for lactation services under the Affordable Care Act, are steps in this direction, although most women around the world still lack access to baby-friendly hospitals [24]. Moreover, there is still a general lack of emphasis on postpartum mothers’ health and well-being, including providing them with timely and extended help in learning to breastfeed throughout the perinatal period [7, 55]. Himbas’ shared cultural model of breastfeeding points to some critical features that can help us to continue moving toward these aims. Their emphasis is on a holistic model of support that centers rest and recuperation for new mothers, as well as the opportunity to learn. In addition to strong norms of support, there is also a core base of shared knowledge about what breastfeeding will be like, likely barriers, and the perception that help is available to overcome those barriers. More focus on breastfeeding as a learned behavior can help us to rethink the ways that we support new mothers to meet their breastfeeding goals.

## Supplementary Material

SUPPLEMENTARY_MATERIALS_eoaf030

## Data Availability

Individualized response data are not publicly available, as I did not have consent to share individualized data. Code for the consensus analysis is available through OSF (link in Supplementary Materials).
