# Supplementary material for: Social learning is critical to breastfeeding success: Evidence from rural Namibian pastoralists
Source: Evol Med Public Health. 2025 Oct 21;13(1):344–54. doi: 10.1093/emph/eoaf030 (PMC12599308; doi:10.1093/emph/eoaf030)
Supplement: SUPPLEMENTARY_MATERIALS_eoaf030 [file supplementary_materials_eoaf030.docx]

**SUPPLEMENTARY MATERIALS**

Social learning is critical to breastfeeding success: evidence from rural Namibian pastoralists

Brooke A. Scelza^1^

^1^ UCLA Department of Anthropology, Los Angeles, CA 90095; bscelza@gmail.com

Supplementary Methods

Code for the consensus analysis is available at: https://osf.io/my48u/?view_only=c3fe39de97474764bd81b3dfc1f4b640

Supplementary Results

Table S1. Percent agreement with consensus statements (n=127)

| **question_number** | **question** | **% Agree** |
| --- | --- | --- |
| **1** | Women find it difficult to breastfeed when they have their first child | 55.9 |
| **2** | Women need to learn how to breastfeed properly | 85.8 |
| **3** | Women need a lot of rest after birth | 98.4 |
| **4** | Breastfeeding is a way for mothers to bond with their babies | 100 |
| **5** | Pain is a normal part of breastfeeding in the beginning | 96.8 |
| **6** | Others should help with women’s work while she learns to take care of her baby | 95.3 |
| **7** | Breastfeeding is easy | 78.7 |
| **8** | It’s ok to ask for help learning to breastfeed if you need it | 85 |
| **9** | Women who are breastfeeding get enough sleep | 17.3 |
| **10** | Women just know how to breastfeed, they don’t need to learn | 70.1 |
| **11** | Women should be able to go back to their normal working conditions about a week after birth | 26.8 |
| **12** | Breastfeeding is stressful | 31 |
| **13** | It’s a mother’s job to teach her daughter to breastfeed | 96.8 |
| **14** | If you feel pain when you are breastfeeding, you are doing it wrong | 37.3 |
| **15** | It’s difficult to find people you can ask when you have questions about breastfeeding | 11.8 |
| **16** | Women are scared to breastfeed in the beginning | 74 |
| **17** | Breastfeeding can be a lonely experience | 55.9 |
| **18** | Breastfeeding is instinctual, it doesn’t need to be learned | 91.3 |
| **19** | You should always know when it’s time to feed your baby | 100 |
| **20** | A woman who doesn’t have help learning to breastfeed will never learn | 44.9 |
| **21** | Women should keep doing all their regular work even when they have a new baby | 49.6 |

Table S2. Participant responses broken out by age

| **question_number** | **question** | **<=20** | **21 to 30** | **31 to 40** | **41 to 50** | **>50** |
| --- | --- | --- | --- | --- | --- | --- |
|  | n | 23 | 43 | 27 | 14 | 20 |
| **1** | Women find it difficult to breastfeed when they have their first child | 56.5 | 58.1 | 48.1 | 71.4 | 50 |
| **2** | Women need to learn how to breastfeed properly | 87 | 83.7 | 92.6 | 71.4 | 90 |
| **3** | Women need a lot of rest after birth | 100 | 95.3 | 100 | 100 | 100 |
| **4** | Breastfeeding is a way for mothers to bond with their babies | 100 | 100 | 100 | 100 | 100 |
| **5** | Pain is a normal part of breastfeeding in the beginning | 100 | 95.2 | 96.3 | 92.9 | 100 |
| **6** | Others should help with women’s work while she learns to take care of her baby | 95.7 | 90.7 | 96.3 | 100 | 100 |
| **7** | Breastfeeding is easy | 65.2 | 74.4 | 85.2 | 92.9 | 85 |
| **8** | It’s ok to ask for help learning to breastfeed if you need it | 87 | 81.4 | 88.9 | 92.9 | 80 |
| **9** | Women who are breastfeeding get enough sleep | 8.7 | 20.9 | 22.2 | 14.3 | 15 |
| **10** | Women just know how to breastfeed, they don’t need to learn | 73.9 | 62.8 | 66.7 | 85.7 | 75 |
| **11** | Women should be able to go back to their normal working conditions about a week after birth | 17.4 | 32.6 | 25.9 | 28.6 | 25 |
| **12** | Breastfeeding is stressful | 30.4 | 26.2 | 40.7 | 28.6 | 30 |
| **13** | It’s a mother’s job to teach her daughter to breastfeed | 91.3 | 95.3 | 100 | 100 | 100 |
| **14** | If you feel pain when you are breastffeeding, you are doing it wrong | 43.5 | 40.5 | 37 | 28.6 | 30 |
| **15** | It’s difficult to find people you can ask when you have questions about breastfeeding | 8.7 | 14 | 7.4 | 21.4 | 10 |
| **16** | Women are scared to breastfeed in the beginning | 69.6 | 79.1 | 77.8 | 64.3 | 70 |
| **17** | Breastfeeding can be a lonely experience | 39.1 | 58.1 | 66.7 | 64.3 | 50 |
| **18** | Breastfeeding is instinctual, it doesn’t need to be learned | 91.3 | 88.4 | 96.3 | 92.9 | 90 |
| **19** | You should always know when it’s time to feed your baby | 100 | 100 | 100 | 100 | 100 |
| **20** | A woman who doesn’t have help learning to breastfeed will never learn | 43.5 | 44.2 | 55.6 | 50 | 30 |
| **21** | Women should keep doing all their regular work even when they have a new baby | 34.8 | 44.2 | 55.6 | 64.3 | 60 |

Table S3. Participant responses broken out by Parity

| **question_number** | **question** | **nulliparous** | **one to three** | **more than three** |
| --- | --- | --- | --- | --- |
|  | n | 17 | 49 | 61 |
| **1** | Women find it difficult to breastfeed when they have their first child | 58.8 | 55.1 | 55.7 |
| **2** | Women need to learn how to breastfeed properly | 82.4 | 85.7 | 86.9 |
| **3** | Women need a lot of rest after birth | 100 | 95.9 | 100 |
| **4** | Breastfeeding is a way for mothers to bond with their babies | 100 | 100 | 100 |
| **5** | Pain is a normal part of breastfeeding in the beginning | 100 | 95.9 | 96.7 |
| **6** | Others should help with women’s work while she learns to take care of her baby | 94.1 | 91.8 | 98.4 |
| **7** | Breastfeeding is easy | 64.7 | 67.3 | 91.8 |
| **8** | It’s ok to ask for help learning to breastfeed if you need it | 94.1 | 75.5 | 90.2 |
| **9** | Women who are breastfeeding get enough sleep | 5.9 | 16.3 | 21.3 |
| **10** | Women just know how to breastfeed, they don’t need to learn | 94.1 | 59.2 | 72.1 |
| **11** | Women should be able to go back to their normal working conditions about a week after birth | 17.6 | 30.6 | 26.2 |
| **12** | Breastfeeding is stressful | 25 | 26.5 | 36.1 |
| **13** | It’s a mother’s job to teach her daughter to breastfeed | 94.1 | 93.9 | 100 |
| **14** | If you feel pain when you are breastffeeding, you are doing it wrong | 35.3 | 37.5 | 37.7 |
| **15** | It’s difficult to find people you can ask when you have questions about breastfeeding | 0 | 16.3 | 11.5 |
| **16** | Women are scared to breastfeed in the beginning | 76.5 | 75.5 | 72.1 |
| **17** | Breastfeeding can be a lonely experience | 47.1 | 57.1 | 57.4 |
| **18** | Breastfeeding is instinctual, it doesn’t need to be learned | 88.2 | 95.9 | 88.5 |
| **19** | You should always know when it’s time to feed your baby | 100 | 100 | 100 |
| **20** | A woman who doesn’t have help learning to breastfeed will never learn | 52.9 | 40.8 | 45.9 |
| **21** | Women should keep doing all their regular work even when they have a new baby | 47.1 | 40.8 | 57.4 |

Table S4. Participant responses broken out by education level

| **question_number** | **question** | **no schooling** | **some schooling** |
| --- | --- | --- | --- |
|  | n | 98 | 27 |
| **1** | Women find it difficult to breastfeed when they have their first child | 58.2 | 48.1 |
| **2** | Women need to learn how to breastfeed properly | 85.7 | 85.2 |
| **3** | Women need a lot of rest after birth | 98 | 100 |
| **4** | Breastfeeding is a way for mothers to bond with their babies | 100 | 100 |
| **5** | Pain is a normal part of breastfeeding in the beginning | 96.9 | 96.3 |
| **6** | Others should help with women’s work while she learns to take care of her baby | 94.9 | 96.3 |
| **7** | Breastfeeding is easy | 77.6 | 81.5 |
| **8** | It’s ok to ask for help learning to breastfeed if you need it | 85.7 | 81.5 |
| **9** | Women who are breastfeeding get enough sleep | 17.3 | 14.8 |
| **10** | Women just know how to breastfeed, they don’t need to learn | 70.4 | 74.1 |
| **11** | Women should be able to go back to their normal working conditions about a week after birth | 27.6 | 18.5 |
| **12** | Breastfeeding is stressful | 28.9 | 37 |
| **13** | It’s a mother’s job to teach her daughter to breastfeed | 97.9 | 92.6 |
| **14** | If you feel pain when you are breastffeeding, you are doing it wrong | 37.1 | 33.3 |
| **15** | It’s difficult to find people you can ask when you have questions about breastfeeding | 12.2 | 11.1 |
| **16** | Women are scared to breastfeed in the beginning | 72.4 | 77.8 |
| **17** | Breastfeeding can be a lonely experience | 58.2 | 44.4 |
| **18** | Breastfeeding is instinctual, it doesn’t need to be learned | 90.8 | 96.3 |
| **19** | You should always know when it’s time to feed your baby | 100 | 100 |
| **20** | A woman who doesn’t have help learning to breastfeed will never learn | 46.9 | 37 |
| **21** | Women should keep doing all their regular work even when they have a new baby | 54.1 | 33.3 |
